# Conserved miRNAs and Their Response to Salt Stress in Wild Eggplant *Solanum linnaeanum* Roots

**DOI:** 10.3390/ijms15010839

**Published:** 2014-01-09

**Authors:** Yong Zhuang, Xiao-Hui Zhou, Jun Liu

**Affiliations:** Institute of Vegetable Crops, Jiangsu Academy of Agricultural Sciences, Nanjing 210014, China; E-Mails: xhzhou1984@sina.com (X.-H.Z.); Kehl_lau@foxmail.com (J.L.)

**Keywords:** salt stress, miRNA, *Solanum linnaeanum*, high-throughput sequencing

## Abstract

The Solanaceae family includes some important vegetable crops, and they often suffer from salinity stress. Some miRNAs have been identified to regulate gene expression in plant response to salt stress; however, little is known about the involvement of miRNAs in Solanaceae species. To identify salt-responsive miRNAs, high-throughput sequencing was used to sequence libraries constructed from roots of the salt tolerant species, *Solanum linnaeanum*, treated with and without NaCl. The sequencing identified 98 conserved miRNAs corresponding to 37 families, and some of these miRNAs and their expression were verified by quantitative real-time PCR. Under the salt stress, 11 of the miRNAs were down-regulated, and 3 of the miRNAs were up-regulated. Potential targets of the salt-responsive miRNAs were predicted to be involved in diverse cellular processes in plants. This investigation provides valuable information for functional characterization of miRNAs in *S. linnaeanum*, and would be useful for developing strategies for the genetic improvement of the Solanaceae crops.

## 1. Introduction

Salt stress is one of the most common abiotic stresses of crops. It was estimated that salt stress may affect half of all arable lands and will be a major factor of agriculture production for the coming decades [1]. Unlike other abiotic stresses, salt stress brings both osmotic stress and ion toxicity to crops. Under salt stress, crops can respond via cascades of molecular networks to change gene expression profile and posttranslational modifications involved in a broad spectrum of biochemical, cellular and physiological processes [2,3]. Therefore, an understanding of the basis of the salt stress response is important for strategies aimed at improving crop tolerance to salt stress.

miRNAs are endogenous non-coding small RNAs that are regulators of gene expression in organisms. They are known to play negative regulatory functions at the post-transcription level by inhibiting gene translation or cleaving target mRNAs via base-pairing their target mRNAs [4–6]. Many investigations indicated that plant miRNAs are involved in various important physiological processes, such as seed germination and root development [7–9]. In addition, increasing evidence has shown that miRNAs play important roles in the response of plants to biotic and abiotic stresses [10]; the expression levels of miRNAs were changed in plants infected with virus and fungus [11–13], and miRNAs were identified to be involved in plant response to abiotic stresses such as temperature [14,15], drought [16,17], metals [18,19], and salt [20–22].

The Solanaceae family includes some agriculturally important crops such as potato (*Solanum tuberosum*), eggplant (*S. melongena*), tomato (*S. lycopersicum*), and pepper (*Capsicum annuum*), and they often suffer from salt stress that can cause reduction of production, especially in greenhouse production. *S. linnaeanum*, which was used to construct a comparative genetic linkage map of eggplant, has tolerance to salt stress [23,24], however, little is known about the mechanism in response to salt stress. Comparative genomic studies revealed that relatively few genome rearrangements and duplications occurred in the evolutionary history of the Solanaceae species [25–28]. Although little information is known about the genomes of *S. linnaeanum* and *S. melongena*, the published data of other plants, especially those from Solanaceae family, may provide sufficient reference.

In the present study, using high-throughput sequencing, a large number of miRNAs and their response to salt stress in *S. linnaeanum* roots are identified and characterized. The results lay the foundation for further investigation and better understanding of the regulatory mechanisms for the plant response to salt stress. In addition, it also provides important information for genetic improvement of Solanaceae crops to salt stress.

## 2. Results and Discussion

### 2.1. Deep Sequencing Results of Small RNAs from *S. linnaeanum* Roots

**Figure 1.**
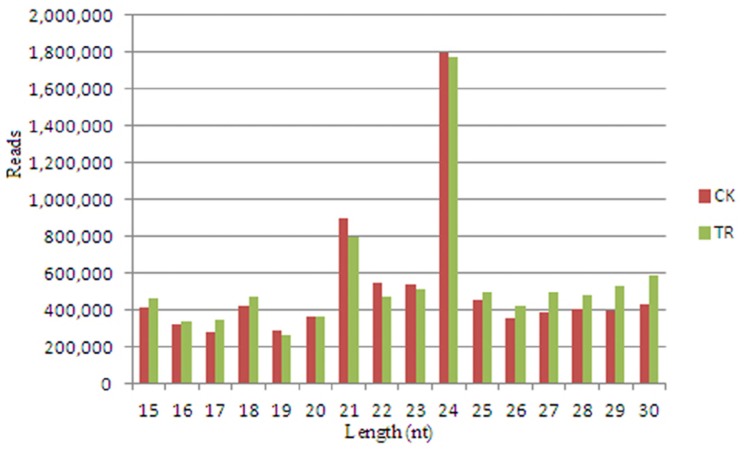
Length distribution of mappable small RNAs in two databases of *S. linnaeanum* roots. TR represents library of NaCl treatment, and CK represents library of control. The number in vertical axis is the total reads of all small RNAs in a certain length.

To identify the miRNAs and their response to salt in *S. linnaeanum*, two small RNA libraries were generated from roots of NaCl-free (CK) and NaCl-treated (TR). Deep sequencing generates 21,284,496 and 13,989,100 raw reads in two libraries. After removal of low-quality and corrupted adapter sequences, 8,462,890 and 8,999,145 mappable reads remain in two libraries. The size distribution of mappable reads is assessed (Figure 1, Table S1). The data show that 24 nt small RNA is the major size class, followed by 21, 23, 30 and 22 nt small RNA. Similar results were reported in some other plant species, such as *Arabidopsis thaliana* [29,30], *Medicago truncatula* [31], *Oryza sativa* [32], *Arachis hypogaea* [33], *Cucumis Sativus* [34], *Nicotiana tabacum* [35], and *Citrus trifoliate* [36].

Because details of *S. linnaeanum* genome are limited, these mappable reads are analyzed with genome information of tomato and other plants. The results show that 5.51% reads of CK and 4.86% reads of TR are mapped to known plant pre-miRNAs in miRbase. Reads from CK (24.17%) and TR (24.29%) are mapped to plant repeats, mRNA, and other RNAs including tRNA, rRNA, snRNA and snoRNA. In addition, some reads that cannot be mapped to pre-miRNAs in miRbase and other RNAs are mapped to tomato genome sequences, and a fraction of them potentially form hairpins. Also, nearly half of these reads have no mapping information (Table 1). To eliminate possible sequencing errors, only those sequences with more than five reads in either of the two libraries are further analyzed.

### 2.2. Conserved miRNAs in *S. linnaeanum* Roots

To identity the conserved miRNAs in *S. linnaeanum* roots, small RNA sequences are mapped to tomato and other plant miRNAs in miRBase. Based on sequence homology (number of mismatch < 3), 98 known miRNAs and 7 miRNAs* are found (Table S2). The majority of these miRNAs are 20–22 nt long, and 56 of them are 21 nt long. These identified conserved miRNAs correspond to 37 families. The number of miRNA members in each known family shows significant divergence. The miR166 family is the largest one with 11 members, and for the family of miR171, miR396, and miR156, each of them has 7, 6, and 5 members respectively. Six families including miR159, miR162, miR167, miR168, miR319, and miR390 contain four members, and the remaining 27 miRNA families contain one to three members.

The read counts of miRNAs in sequencing libraries can be used as an index to estimate their relative abundance. In this study, the read counts differ among the miRNAs, which indicate that their expressions varied. Counting redundant miRNA reads reveals that 18 out of 98 known miRNAs and 2 miRNAs* are represented by more than 1000 reads in both libraries, and 5 of them, sli-miR166e (201,378 reads), sli-miR2911c (48,948 reads), sli-miR396d (29,823 reads), sli-miR166f (29,594 reads), and sli-miR403a (28,676 reads) are the most frequent. In addition, sequence analysis shows that the relative abundance of certain member within the miRNA families varies greatly, suggesting functional divergence within the family. For instance, reads of the sli-miR166 family vary from 10 reads (sli-miR166k) to 201,378 reads (sli-miR166e). Similar results are observed in some other miRNA families, such as sli-miR396 (7-29,823 reads) and sli-miR2911 (256-48,948 reads). The above results indicate the different expression levels of different miRNAs in roots, and may be the result of tissue specific or developmental expression.

### 2.3. Validation of miRNAs in *S. linnaeanum* Roots

**Figure 2.**
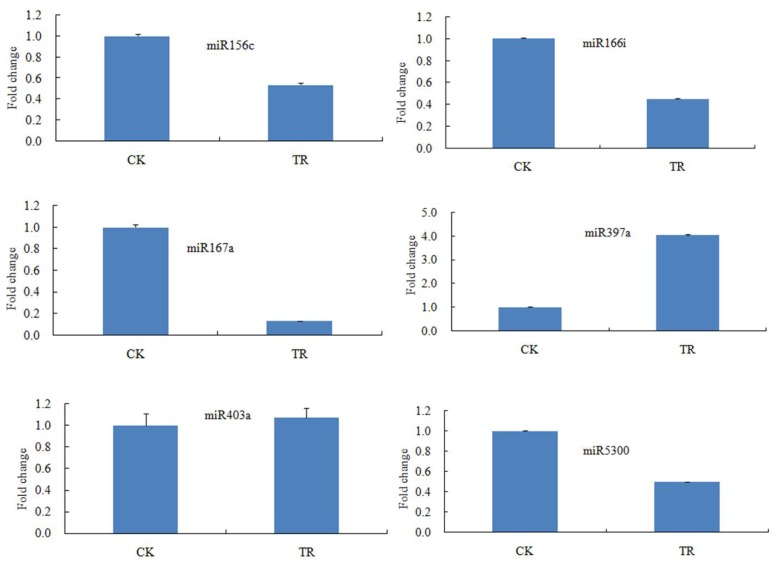
Validation of selected miRNAs in roots by qRT-PCR. The data are the average of three qRT-PCR replicates for each sample from three biological repeats. Small nuclear RNA U6 is used as an internal reference. Error bars indicate one standard deviation of three different biological replicates. The expression changes of six miRNAs detected by qRT-PCR are consistent with the Illumina sequencing results.

To verify the results of RNA sequencing and bioinformatics analysis, six miRNAs (sli-miR156c, sli-miR166i, sli-miR167a, sli-miR397a, sli-miR403a and sli-miR5300) are selected randomly for validation by qRT-PCR. According to the Illumina sequencing results, these miRNAs are four down-regulated miRNAs, one up-regulated miRNA and one no responsive miRNA. As shown in the Figure 2 and Table S3, the expression changes detected by qRT-PCR for 4 miRNAs (sli-miR156c, sli-miR166i, sli-miR397a and sli-miR403a) are similar to the results of Illumina sequencing. For sli-miR167a and sli-miR5300, the results have small differences, but they all show down regulation. This may be induced by sequencing error or sampling difference. Above results suggest that miRNAs and their expression changes under NaCl stress have been successfully discovered from *S. linnaeanum* roots by Illumina sequencing.

### 2.4. NaCl-Responsive miRNAs in *S. linnaeanum* Roots

A deep sequencing approach can be used as a powerful tool for profiling miRNA expression [15,31]. The changes in the frequency of miRNAs between the NaCl-treated and control libraries might indicate that their expression is regulated in response to NaCl stress. To minimize noise and improve accuracy, only the 18–24 nt miRNAs with normalized sequence reads over 10 in at least one library are selected for comparison. miRNAs with log_2_(TR/CK) > 1 and *p* < 0.05 are designated as up-regulated. Similarly, miRNAs with log_2_(TR/CK) < −1 and *p* < 0.05 are designated as down-regulated. As showed in Table 2, under the stress of NaCl treatment, 11 miRNAs belonging to eight families are down-regulated, and three miRNAs belonging to three families are up-regulated. The above results indicate that the number of NaCl-induced down-regulated miRNAs is more than that of up-regulated miRNAs.

To understand the potential functions of NaCl-responsive miRNAs, 31 target genes (Table S4) for these miRNAs are predicted, and the representative results are listed in Table 2. These genes were reported to be involved in many plant physiological processes, such as plant development, metabolism, and defense. Interestingly, different members in a miRNA family may target the same or different genes. For example, both sli-miRNA156b and sli-miRNA156c can target genes encoding squamosa promoter-binding protein-like, which indicates that they are functionally conservative. The same results are observed for sli-miR171b and sli-miR171e, and they all target the gene encoding scarecrow transcription factor family protein. However, for sli-miR167a and sli-miR167b, which can target different genes, their functions may be differentiated by sequence variation.

As *S. linnaeanum* is salt-tolerant, these salt responsive miRNAs may play an important role for salt tolerance. Some miRNAs, such as *Zea mays* miR166, miR159, miR156 and miR319, and Arabidopsis miR393, miR397b, and miR402, have been reported to show altered expression profile under salt stress [21,37]. In the present study, one of the up-regulated miRNA, sli-miR397a, is predicted to target a laccase gene, which was reported to reduce root growth under dehydration [38]. Similarly, another up-regulated miRNA, sli-miR166d, is predicted to target a DNA repair protein RAD4 family gene which was previously found as a key repair factor that directly recognizes DNA damage and initiates DNA repair, and recently it was found to regulate protein turnover at a postubiquitylation step [39]. Because of the salt tolerance of *S. linnaeanum*, it is possible that the roots do not suffer with serious injuries such as reduced growth and DNA damage. Therefore, the two potential targets do not need to show high expression to alleviate the injuries. However, further investigations are needed to confirm the above hypothesis.

Unlike the targets of up-regulated miRNAs *in S. linnaeanum*, some of the down-regulated miRNAs target mRNAs of transcription factors, indicating an upstream regulation of miRNAs during the response to salt stress. sli-miR171b and sli-miR171e are predicted to target a scarecrow transcription factor gene which was reported to be involved in ground tissue formation in Arabidopsis root [40]. sli-miR172a is predicted to target an Floral homeotic protein APETALA2 gene. SlAP2a, the true ortholog of AP2 in tomato has been found to control fruit ripening via regulation of ethylene biosynthesis and signaling [41]. However, the role of AP2 in response to salt stress has not been described in detail. sli-miR319a is predicted to target a TCP family transcription factor gene which was reported to play a pivotal role in the control of morphogenesis of shoot organs by negatively regulating the expression of boundary-specific genes in Arabidopsis [42]. The above results indicate that the function involved in the response to salt stress of these potential targets needs to be explored in depth. The identification of salt-responsive miRNAs that target these genes may suggest additional roles for the defense against salt stress.

## 3. Experimental Section

### 3.1. Plant Materials and NaCl Treatment

A wild eggplant species, *S. linnaeanum* (PI388846) is used in this study. The seeds are surface-sterilized with 70% ethanol, and allowed to germinatein 30 °C. The uniform germinated seeds are sown in pots containing commercial nursery substrate. The seedlings are grown in an incubator with a 16 h photoperiod at a temperature regime of 25 °C. When the seedlings develop five true leaves, uniform seedlings are picked out and irrigated with 150 mM NaCl for salt treatment or the distilled water as a control. The roots of the NaCl treated and control plants are harvested after 24 h. The collected roots are pooled with ten plants and immediately frozen in liquid nitrogen for RNA extraction.

### 3.2. Small RNA Library Construction and Sequencing

Total RNA is extracted with the Total RNA Purification Kit (Norgen Biotek, Thorold, Canada and treated with *DNase I* according to the manufacturer’s instructions. The small RNA libraries are constructed using the Truseq™ Small RNA Preparation kit (Illumina, San Diego, CA, USA). The purified cDNA library from 15 to 32 nt small RNAs is used for cluster generation on Illumina’s Cluster Station and then sequenced on Illumina GAIIx (San Diego, CA, USA). Raw sequencing reads are obtained using Illumina’s Sequencing Control Studio software version 2.8 (SCS v2.8, San Diego, CA, USA) following real-time sequencing image analysis and base-calling by Illumina’s Real-Time Analysis version 1.8.70 (RTA v1.8.70).2.1.1 (Illumina, San Diego, CA, USA).

### 3.3. Analysis of Small RNA Sequencing Data

A proprietary pipeline script, ACGT101-miR v4.2 (LC Sciences, Houston, TX, USA), is used for sequencing data analysis. The “impurity” reads due to sample preparation, sequencing chemistry and processes, and the optical digital resolution of the sequencer detector are removed. Those remaining sequences are grouped by families (unique sequences). Thereafter, families that match known plant repeats, mRNA, rRNAs, tRNAs, snRNAs, and snoRNAs were removed. The remaining unique sequences are mapped to known plant miRNAs from miRBase and Pre-miRBase (Version 17.0, ftp://mirbase.org/pub/mirbase/CURRENT, University of Manchester, Manchester, UK) and *S. lycopersicum* genome database (PlantGDB, ftp://ftp.plantgdb.org/download/Genomes/SlGDB/ITAG2_genomic.fasta).

The number of read copies from each sample is tracked during mapping and normalized for comparison. The normalization of sequence counts in each sample is achieved by dividing the counts by a library size parameter of the corresponding sample. The library size parameter is a median value of the ratio between the counts a specific sample and a pseudo-reference sample. A count number in the pseudo-reference sample is the count geometric mean across two samples. For miRNA expression analysis, *p* value calculation is performed with the method introduced by Audic and Claverie [43].

### 3.4. miRNA Validation by Quantitative Real-Time PCR

The identified *S. linnaeanum* miRNAs are validated by using quantitative real time PCR (qRT-PCR). In this study, six conserved miRNAs (sli-miR156c, sli-miR166i, sli-miR167a, sli-miR397a, sli-miR403a and sli-miR5300) are validated. Total RNA is isolated from roots of CK and TR, which are samples of parallel experiments for RNA sequencing. For determination of miRNA expression, RNAs are reverse-transcribed by miScript II Reverse Transcription Kit (Qiagen, Germantown, MD, USA), which adds a poly (A) tail to the 3′-end of miRNA and with transcription led by a known oligo-dT ligate. SuperReal PreMix (SYBR Green, TIANGEN, Beijing, China) is used for qRT-PCR. Small nuclear RNA U6 is used as an internal reference. The primers for the 6 miRNAs are universal primers (QIAGEN, Germantown, MD, USA) and corresponding miRNA sequences. qRT-PCR experiments are performed on Roche LightCycler 480 II. PCR program is set as: (1) 95 °C, 15 min; (2) 95 °C, 10 s, thereafter 60 °C, 30 s, 40 cycles. All reactions are run in three replicates for each sample from three biological repeats.

### 3.5. Prediction of miRNA Target Genes

The putative target sites of miRNA are identified using the psRNATarget program (http://plantgrn.noble.org/psRNATarget/) with default parameters [44]. Because there is not enough genome information for *S. melongena* and *S. linnaeanum*, the database of tomato *S. lycopersicum* is used as the sequence library for target search.

## 4. Conclusions

In this study, by using high-throughput sequencing and taking advantage of the genome information of other plants, 98 known miRNAs were discovered in *S. linnaeanum* roots, and 14 of them show response to salt stress. The potential targets of the identified salt responsive miRNAs are also predicted based on sequence homology search. However, the further investigation for the function of potential target genes still needs to be performed. As more salt tolerance related miRNAs are confirmed, artificial miRNA will be a powerful tool to create elite plant germplasm with salt tolerance.

## Supplementary Information



## Figures and Tables

**Table 1.** Statistical analysis of sequencing reads in the two libraries.

Category	CK	Percent (%)	TR	Percent (%)
Raw reads	13,989,100		21,284,496	
Mappable reads	8,462,890	100.00	8,999,145	100.00
Mapped to miRNA	466,136	5.51	437,144	4.86
Mapped to mRNA	723,297	8.55	793,475	8.82
Mapped to RFam	1,315,886	15.55	1,387,942	15.42
Mapped to Repbase	5,674	0.07	4,745	0.05
Mapped to genome	1,763,934	20.84	2,063,801	22.93
No hit	4,187,963	49.49	4,312,038	47.92

**Table 2.** NaCl-responsive miRNAs and their targets. The value of TR/CK is the ratio between normalized count from library TR and CK.

miRNA	Log_2_(TR/CK)	*p* value	Predicted target	Putative function of target
sli-miR156b	−1.01	2.46 × 10^−127^	SGN-U325281	Squamosa promoter-binding protein
sli-miR156c	−1.18	2.72 × 10^−52^	SGN-U317176	Squamosa promoter-binding protein
sli-miR162b	−1.25	8.35 × 10^−99^	Solyc10g005130.2.1	Ribonuclease 3-like protein 3
sli-miR164c	1.03	3.57 × 10^−16^	SGN-U327571	Lipase-related
sli-miR166d	1.97	1.02 × 10^−45^	Solyc11g011150.1.1	DNA repair protein Rad4 family
sli-miR167a	−1.45	4.26 × 10^−166^	SGN-U313907	Annexin 1
sli-miR167b	−1.25	5.57 × 10^−15^	Solyc03g095940.1.1	LOB domain family protein
sli-miR171b	−1.18	1.89 × 10^−47^	SGN-U333058	Scarecrow transcription factor family protein
sli-miR171e	−1.08	1.24 × 10^−12^	SGN-U333058	Scarecrow transcription factor family protein
sli-miR172a	−1.66	1.94 × 10^−44^	SGN-U563871	Floral homeotic protein APETALA2
sli-miR319a	−1.07	2.28 × 10^−28^	SGN-U31990	TCP family transcription factor
sli-miR397a	1.91	1.04 × 10^−43^	SGN-U327694	Laccase
sli-miR399b	−1.14	9.82 × 10^−14^	Solyc03g031410.1.1	Unknown Protein
sli-miR5300	−1.92	1.55 × 10^−155^	SGN-U336733	CC-NBS-LRR protein
