# Supplementary material for: Conserved miRNAs and Their Response to Salt Stress in Wild Eggplant Solanum linnaeanum Roots
Source: Int J Mol Sci. 2014 Jan 9;15(1):839–49. doi: 10.3390/ijms15010839 (PMC3907842; doi:10.3390/ijms15010839)
Supplement: Supplementary file 1 [file ijms-15-00839-s001.pdf]

Supplementary Information

Table S1. Length distribution of mappable reads.

Length	CK		TR	
	Reads	Percent (%)	Reads	Percent (%)
15	415,544	5.00	460,556	5.21
16	318,260	3.83	339,920	3.85
17	284,070	3.42	344,876	3.90
18	422,230	5.08	472,581	5.35
19	288,068	3.47	261,274	2.96
20	366,351	4.41	366,209	4.15
21	894,463	10.76	799,295	9.05
22	548,725	6.60	474,535	5.37
23	536,209	6.45	515,376	5.83
24	1,798,032	21.64	1,772,935	20.07
25	452,080	5.44	499,965	5.66
26	356,904	4.29	423,854	4.80
27	392,512	4.72	497,906	5.64
28	403,207	4.85	477,987	5.41
29	400,644	4.82	534,469	6.05
30	433,305	5.21	590,820	6.69

Table S2. Conserved miRNAs in *S. linnaeanum* roots.

Name	Mature miRNA	ML	CK reads	TR reads	Log_2(TR/CK)
sli-miR156a	GCTCACTGCTCTATCTGTCACC	22	37	36	−0.02
sli-miR156b	TGACAGAAGAGAGTGAGCAC	20	55	27	−1.01
sli-miR156c	TTGACAGAAGATAGAGAGCAC	21	284	126	−1.18
sli-miR156d	GCTCTCTATGCTTCTGTCATC	21	3	5	0.64
sli-miR156e	GCTCACTTCTCATTCTGTCAGC	22	4	6	0.45
sli-miR159a	TTTGGATTGAAGGGAGCTCTT	21	43	57	0.42
sli-miR159b	TTTGGATTGAAGGGAGCTCTA	21	3,932	5,618	0.51
sli-miR159c	AGCTGCTGACCTATGGATTCC	21	2,052	1,664	−0.30
sli-miR159d	TTTGGATTGAAGGGAGCTCTAC	22	5	1	−2.14
sli-miR160a	TGCCTGGCTCCCTGTATGCCA	21	158	134	−0.24
sli-miR160b	GCGTATGAGGAGCCAAGCATA	21	39	27	−0.52
sli-miR162a	TCGATAAACCTCTGCATCCAGA	22	14	11	−0.38
sli-miR162b	TCGATAAACCTCTGCATCCAGT	22	47	20	−1.25
sli-miR162c	TCGATAAACCTCTGCATCCAG	21	4,448	2,777	−0.68
sli-miR162d	GGAGGCAGCGGTTCATCGATC	21	116	83	−0.49
sli-miR164a	TGGAGAAGCAGGGCACGTGCAC	22	11	6	−0.93
sli-miR164b	TGGAGAAGCAGGGCACGTGCA	21	265	177	−0.58
sli-miR164c	TGGAGAAGCAGGGCACATGCTG	22	5	11	1.03
sli-miR165a	CCGGACCAGGCTTCATTCCCC	21	293	374	0.35
sli-miR165b	ACTCGGACCAGGCTTCA	17	3	11	2.03
sli-miR166a	CCTCGGACCAGGCTTCATTCC	21	66	77	0.23
sli-miR166b	TCGGACCAGGCTTCATTCCT	20	63	45	−0.49
sli-miR166c	ATCTCGGACCAGGCTTCATTCCCC	24	12	12	0.00

Table S2. *Cont.*

Name	Mature miRNA	ML	CK reads	TR reads	Log_2(TR/CK)
sli-miR166d	TCTCGGACCAGGCTCCATTCC	21	7	27	1.97
sli-miR166e	TCGGACCAGGCTTCATTCCCC	21	88,288	113,090	0.36
sli-miR166f	GGAATGTTGTCTGGCTCGAGG	21	16,303	13,291	−0.29
sli-miR166g	TCGGACCAGGCTTCATTCCTC	21	2,732	2,877	0.07
sli-miR166h	TCTCGGANCAGGCTTCATTCC	21	776	943	0.28
sli-miR166i	TCGGACCAGGCTTCATTCCCCA	22	9	5	−1.01
sli-miR166j	TCTCGGATCAGGCTTCATTCC	21	8	6	−0.40
sli-miR166k	AGGCGTCGGACCAGGCTTCATTCC	24	5	5	−0.14
sli-miR167a	TGAAGCTGCCAGCATGATCTGG	22	740	271	−1.45
sli-miR167b	GATCATGTGGTAGCCTCACC	20	11	5	−1.25
sli-miR167c	TTGAAGCTGCCAGCATGATCTG	22	14	9	−0.55
sli-miR167d	TGAAGCTGCCAGCATGATCT	20	110	117	0.08
sli-miR168a	TCGCTTGGTGCAGGTCGGGAA	21	1,145	1,163	0.02
sli-miR168b	TCGCTTGGTGCAGGTCGGGA	20	122	228	0.90
sli-miR168c	TTGCATCAACTGAATCGGAGA	21	24	19	−0.36
sli-miR168d	TCTAATTCGCTTGGTGCAGGT	21	18	26	0.51
sli-miR169a	TTTGATAGCCAAGGATGACT	20	12	9	−0.40
sli-miR169b	TAGCCAAGGATGACTTGCCT	20	2	6	1.45
sli-miR169c	TGGATTGCAGCCAAGGATGAC	21	1	5	2.62
sli-miR171a	CGATATTGGTGAGGTTCAATC	21	13	12	−0.14
sli-miR171b	TTGAGCCGCGTCAATATCTCT	21	27	12	−1.18
sli-miR171c	CGATGTTGGTGAGGTTCAATC	21	16	27	0.72
sli-miR171d	TGAGCCGAACCAATATCACTC	21	32	56	0.82
sli-miR171e	TTGAGCCGCGCCAATATCACT	21	10	5	−1.08
sli-miR171f	TGATTGAGCCGTGCCAATATC	21	8	6	−0.40
sli-miR171g	TAATTTGATTGAGCCGTGCC	20	1	5	2.45
sli-miR172a	GGAATCTTGATGATGCTGCAT	21	27	9	−1.66
sli-miR172b	AGAATCTTGATGATGCTGCAT	21	5	1	−3.14
sli-miR172c	GTAGCATCATCAAGATTCATA	21	3	7	1.45
sli-miR305	CTGAAGTGTTTGGGGGAACTC	21	5	2	−1.55
sli-miR319a	TTGGACTGAAGGGAGCTCCTT	21	17	8	−1.07
sli-miR319b	TGTGAATGATGCGGGAGATAA	21	25	25	−0.02
sli-miR319c	CTTGGACTGAAGGGAGCTCCC	21	420	603	0.52
sli-miR319d	TTGCTGCCGACTCATTCATCCA	22	79	110	0.48
sli-miR390a	AAGCTCAGGAGGGATAGCGCC	21	73	58	−0.32
sli-miR390b	AAGCTCAGGAGGGATAGCACC	21	241	182	−0.41
sli-miR390c	CGCTATCCATCCTGAGTTCT	20	5	0	−12.33
sli-miR390d	TATAGCGCTATCCATCCTGAG	21	7	5	−0.55
sli-miR394	TTTGGCATTCTGTCCACCTCC	21	13	12	−0.14
sli-miR396a	TCCACAGGCTTTCTTGAACTG	21	18	14	−0.36
sli-miR396b	TTCCACAGCTTTCTTGAAC	19	12	4	−1.49
sli-miR396c	TTCCACAGCTTTCTTGAACTT	21	570	503	−0.18
sli-miR396d	CAGCTTTCTTGAACTG	16	9,365	20,458	1.13
sli-miR396e	TTCCACAGCTTTCTTGAACTTTT	23	9	8	−0.14

Table S2. *Cont.*

Name	Mature miRNA	ML	CK reads	TR reads	Log_2(TR/CK)
sli-miR396f	CAGTTCANTAAAGCTGTGGGA	21	6	1	−2.36
sli-miR397a	TCATTGAGTGCAGCGTTGATG	21	7	26	1.91
sli-miR397b	ATTGAGTGCAGCGTTGATGAC	21	39	51	0.42
sli-miR398	TGTGTTCTCAGGTCGCCCCTG	21	2	6	1.45
sli-miR399a	TGCCAAAGGAGAGTTGCCCTG	21	13	9	−0.46
sli-miR399b	GGGCTACACTCTATTGGCATG	21	10	5	−1.14
sli-miR403a	TTAGATTCACGCACAAACTCG	21	14,152	14,524	0.04
sli-miR403b	TTAGATTCACGCACAAACT	19	57	63	0.16
sli-miR408a	TGCACTGCCTCTTCCCTGGCT	21	221	189	−0.22
sli-miR408b	TGCACTGCCTCTTCCCTGGCTT	22	22	13	−0.79
sli-miR482a	TTTCCAATTCCACCCATTCCTA	22	194	316	0.71
sli-miR482b	GATGATGATGATGATGA	17	11	6	−0.72
sli-miR894	TCACGTCGGGTTCACCA	17	1,874	1,787	−0.07
sli-miR1310a	CTCGACCTATTCTCAAACTTTAAATA	26	65	83	0.35
sli-miR1310b	CCCTCGACCTATTCTCAAACTTTAAATA	28	49	36	−0.43
sli-miR1446	TTTCTGAACTCTCTCCCTCAA	21	5	1	−3.01
sli-miR1919	TGTCGCAGATGACTTTCGCCC	21	13	14	0.08
sli-miR2911a	CCCAGTCCCGAACCCG	16	1,292	1,026	−0.33
sli-miR2911b	AACCCGTCGGCTGTCGG	17	104	151	0.54
sli-miR2911c	CGGTGGACTGCTCGAGCTGCT	21	19,905	29,043	0.55
sli-miR2916a	AACCATAAACGATGCCGACC	20	735	843	0.20
sli-miR2916b	TCCTAGTCTCAACCATAAACGATGCCGACC	30	268	267	0.00
sli-miR5072	CCCCAGCGGAGTCGC	15	15	28	0.86
sli-miR5083a	AGACTACAATTATCTGATCA	20	12	7	−0.72
sli-miR5083b	CAATGGATCCTTCTGAGCCT	20	58	49	−0.25
sli-miR5139	AACCTGGCTCTGATACCA	18	174	113	−0.62
sli-miR5141	AGACCCGACGCGACTGACAGA	21	15	9	−0.72
sli-miR5300	CCAGTCCAGGCATTCCAAC	19	75	20	−1.92
sli-miR5301a	TGTGGGTGGGGTGGAAAGATT	21	158	117	−0.43
sli-miR5301b	TCTTTCCTACTCCTCCCATACC	21	1,989	3,707	0.90
sli-miR5303	GCTCGGACTCTCCAAAAATGT	21	5	0	−12.33
sli-miR157 *	GCTCTCTATTCTTCTGTCATC	21	13	5	−1.46
sli-miR165a *	GGAATGTTGTCTGGCTCGAC	20	55	43	−0.34
sli-miR166a *	GGAATGTTGTCTGGCTCGGGG	21	7	16	1.25
sli-miR166e *	GGAATGTTGTTTGGCTCGAGG	21	1,534	879	−0.80
sli-miR168b *	CCCGCCTTGCATCAACTGAAT	21	2,250	2,557	0.19
sli-miR3630 *	TGGGAATCTCTCTGATGCAC	20	33	33	0.03
sli-miR396a *	GTTCAATAAAGCTGTGGGAAG	21	322	310	−0.05

ML, Length of mature miRNA; The value of TR/CK is the ratio between normalized reads from library TR and CK; "+"in Log_2(TR/CK) means up-regulation; and "−"in Log_2(TR/CK) means down-regulation.

Table S3. The Ct values of qRT-PCR.

	Repeat	U6	miR156C	miR 166i	miR 167a	miR 397a	miR 403a	miR 5300
CK	I	30.17	31.92	31.20	32.86	33.67	32.32	32.19
		29.96	32.74	30.82	32.74	33.42	31.12	32.07
		29.96	32.92	30.65	32.81	32.47	31.54	32.73
	II	29.98	32.12	31.25	32.86	33.45	32.00	32.18
		30.14	32.58	30.72	32.84	33.55	31.59	32.11
		29.94	32.78	30.65	32.79	32.65	31.89	32.63
	III	30.05	32.42	31.32	32.76	33.63	31.23	32.15
		29.97	32.57	30.83	32.73	33.58	31.56	32.27
		30.09	32.69	30.62	32.82	33.47	31.78	32.62
TR	I	30.68	33.33	32.35	35.59	31.71	31.85	33.04
		30.59	34.03	32.94	36.68	31.09	31.93	34.79
		30.64	34.72	32.67	36.78	31.63	31.45	34.02
	II	30.55	34.32	32.30	35.63	31.54	32.02	33.17
		30.62	33.89	32.89	36.64	31.29	31.6	34.44
		30.70	33.72	32.67	36.71	31.51	31.44	34.20
	III	30.71	34.13	32.41	35.67	31.53	31.54	33.07
		30.62	33.89	32.98	36.62	31.57	32.07	34.75
		30.64	34.32	32.66	36.82	31.47	31.43	34.05

Table S4. Predicted targets of NaCl-responsive miRNAs.

miRNA	Predicted target
sli-miR156b	SGN-U325281, SGN-U324312, SGN-U342074, SGN-U323360
sli-miR156c	SGN-U317176, SGN-U317177, SGN-U313540, SGN-U319735
sli-miR162b	Solyc10g005130.2.1
sli-miR164c	SGN-U327571, SGN-U326326
sli-miR166d	Solyc11g011150.1.1
sli-miR167a	SGN-U313907, SGN-U318676
sli-miR167b	Solyc03g095940.1.1
sli-miR171b	SGN-U333058, SGN-U343385
sli-miR171e	SGN-U333058, SGN-U343385
sli-miR172a	SGN-U314859, SGN-U314861, SGN-U325104
sli-miR319a	SGN-U319907, SGN-U324731, SGN-U323397
sli-miR397a	SGN-U327694, SGN-U330286, SGN-U312405
sli-miR399b	Solyc03g031410.1.1
sli-miR5300	SGN-U324648, SGN-U336733

© 2014 by the authors; licensee MDPI, Basel, Switzerland. This article is an open access article distributed under the terms and conditions of the Creative Commons Attribution license (http://creativecommons.org/licenses/by/3.0/).
